# Autonomic control over myocardial function in vitro: Evaluation of current and future clinical therapies in a nonclinical setting

**DOI:** 10.14814/phy2.15511

**Published:** 2022-11-16

**Authors:** Michiel Blok, Monique R. M. Jongbloed, Bastiaan J. D. Boukens

**Affiliations:** ^1^ Department of Medical Biology Amsterdam University Medical Center Amsterdam The Netherlands; ^2^ Department of Anatomy & Embryology Leiden University Medical Center Leiden The Netherlands; ^3^ Department of Cardiology Leiden University Medical Center Leiden The Netherlands; ^4^ Department of Physiology Maastricht University Maastricht The Netherlands

Many aspects of heart function, including heart rate and contractility, are under continuous influence of the autonomic nervous system. The autonomic nervous system plays an important role in cardiac physiology by fine‐tuning cardiac function in such a way that it meets the organism's behavior. With respect to its function and structure, the autonomic nervous system can be subdivided into a sympathetic and parasympathetic component of which the combined output is reflected by the sympathovagal balance (Ardell & Armour, 2016). In rest, sympathovagal balance shifts toward parasympathetic predominance resulting in, for example, a slower heart rate. Reversely, during exercise there is a shift toward sympathetic predominance resulting in higher heart rates and increased contractility enabling high cardiac outputs. Although in clinical practice, neuromodulatory strategies show real promise in sustaining heart function, research into improvement of current and development of new therapeutic strategies is crucial for better management and treatment of the growing number of heart failure patients (Shivkumar et al., 2016).

Heart failure, defined by an inability of the heart to provide sufficient cardiac output to meet the oxygen consumption demands of the vital organs (including the heart itself), is associated with severe symptoms, such as dyspnoea and edema, as well as other cardiac sequelae such as arrhythmias. Another sequel of heart failure is a shift toward sympathetic predominance and decreased vagal activity. Normally, increased sympathetic activity enhances cardiac output by stimulation of myocardial β‐adrenergic signaling predominantly through the catecholamine neurotransmitters epinephrine and norepinephrine. Activation of the cardiomyocyte adrenergic receptor leads to phosphorylation of intracellular signaling mediators, ion channels and Ca^2+^ pumps, altogether contributing to elevated Ca^2+^ levels in the cardiomyocyte cytoplasm and consequently increased contractility. In chronic heart failure, maladaptive remodeling occurs and leads to sympathetic dysfunction with adverse effects including abnormal Ca^2+^ cycling in the failing myocardium resulting in impaired myocardial contractility, myocardial necrosis, and an increased risk of developing atrial and ventricular arrhythmias, the latter of which may cause sudden cardiac death (Kaye et al., 1995). Therapeutic strategies that can effectively enhance cardiac contractility in heart failure are in need.

One of the current approaches that aims to improve ejection fraction through enhancing ventricular contractility includes cardiac contractility modulation (CCM), a device‐based therapy for moderate to severe heart failure that involves application of relatively high‐voltage, long‐duration, electrical signals to the right ventricular septal wall during the absolute refractory period. Instead of eliciting a new contraction, CCM influences the biology of the failing myocardium likely through effects that induce changes in gene expression and intracellular Ca^2+^ levels (Blinova et al., 2014; Feaster et al., 2021; Imai et al., 2007). β‐adrenergic receptor blockade can even further improve ejection fraction in a synergistic manner, indicating that there is an important adrenergic signaling component to the mechanism that CCM therapy acts upon (Imai et al., 2007).

Induced pluripotent stem cell (iPSC) technology offers a promising, clinically relevant platform for discovery and screening of new therapeutic strategies. Besides their human origin, the pluripotent nature of these cells allows differentiation into virtually any organ‐specific cell type. While most of current iPSC platforms focus solely on the major cell types that reconstitute the heart, only few platforms appear to include neurons. In this issue of *Physiological Reports*, Narkar et al. introduce a novel human, iPSC‐based in vitro neurocardiac coculture (*iv*NCC) platform for nonclinical assessment of CCM. Using this platform, Narkar et al. demonstrated that motor neurons significantly contribute to CCM‐modulated stimulation on cardiomyocyte contractility, underscoring the significance of neurocardiac interplay in CCM therapy and the study of neurocardiac safety and toxicity. One of the major advantages of the *iv*NCC model is its high reproducibility thanks to its utilization of commercially available human iPSC‐derived cardiomyocytes and motor neurons. However, like the authors mention, the cardiac autonomic nervous system is complex and not built up by just a single type of motor neuron but rather by a multitude of neuronal subtypes that are functionally diverse. Given the current evidence that CCM effects are influenced by neurocardiac interplay, one may argue that sympathetic and parasympathetic neurons would influence and modulate CCM effects in a differential and perhaps opposite manner.

Although the study of either atrial or ventricular arrhythmias determines the cardiomyocyte subtype of choice by itself, it is here that also the subtype of neuron in coculture becomes of relevance. For instance, sympathetic hyperactivity during myocardial ischemia has shown to be proarrhythmic (Boukens et al., 2021). Importantly, while some ventricular arrhythmias can be prevented through vagal nerve stimulation (Vanoli et al., 1991), the opposite effect holds true for atrial fibrillation to the extent that it may even serve as a trigger of this same arrhythmia (Tan et al., 2008). Overall, a more complete definition of the cardiomyocyte and neuronal subtypes used to establish the *iv*NCC model would solidify the current findings.

The current *iv*NCC model could be regarded an early precursor to any future iPSC‐based neurocardiac coculture model tailored for the study of specific cardiac disease‐associated phenomena. In addition, iPSC technology offers the possibility of generating iPSCs from patients who carry specific disease‐causing genetic mutations and variants of interest. Employing patient‐derived iPSCs would allow the study of functional consequences of their genetic background on neurocardiac interplay and how this relates to the effectiveness of therapies such as pharmaceutical therapy and CCM. Moreover, correcting these genetic alterations using CRISPR‐Cas9 allows the generation of isogenic control lines, which subsequently can be used to compare the effect and thereby the contribution of the genetic alteration in either cardiomyocyte or neuron individually while in coculture.

In conclusion, the findings by Narkar et al. not only demonstrate the utility of their *iv*NCC model in assessing the current CCM modality, but moreover urges developers of future cardiac in vitro models to consider including neurons as a potentially crucial, additional cell type to study cardiac disease. Given the relevance of iPSC models as a human platform for disease modeling, we expect inclusion of a neuronal component not only to benefit the assessment of current and new therapies for cardiac diseases but also to improve scientific understanding of the neuronal contribution to cardiac biology and pathology as a whole.

## CONFLICT OF INTEREST

The authors have no conflicts of interest to disclose.
